# Supplementary material for: Physicochemical Stability of Nab-Paclitaxel (Pazenir) Infusion Dispersions in Original Glass Vials and EVA Infusion Bags
Source: Pharmaceutics. 2024 Oct 26;16(11):1372. doi: 10.3390/pharmaceutics16111372 (PMC11597360; doi:10.3390/pharmaceutics16111372)
Supplement: Supplementary file 1 [file pharmaceutics-16-01372-s001.zip › pharmaceutics-3170362-supplementary.pdf]

pharmaceutics

MDPI

Article

Supplementary Materials: Physicochemical Stability of Nab-Paclitaxel (Pazenir) Infusion Dispersions in Original Glass Vials and EVA Infusion Bagss

Helen Linxweiler, Judith Thiesen and Irene Krämer

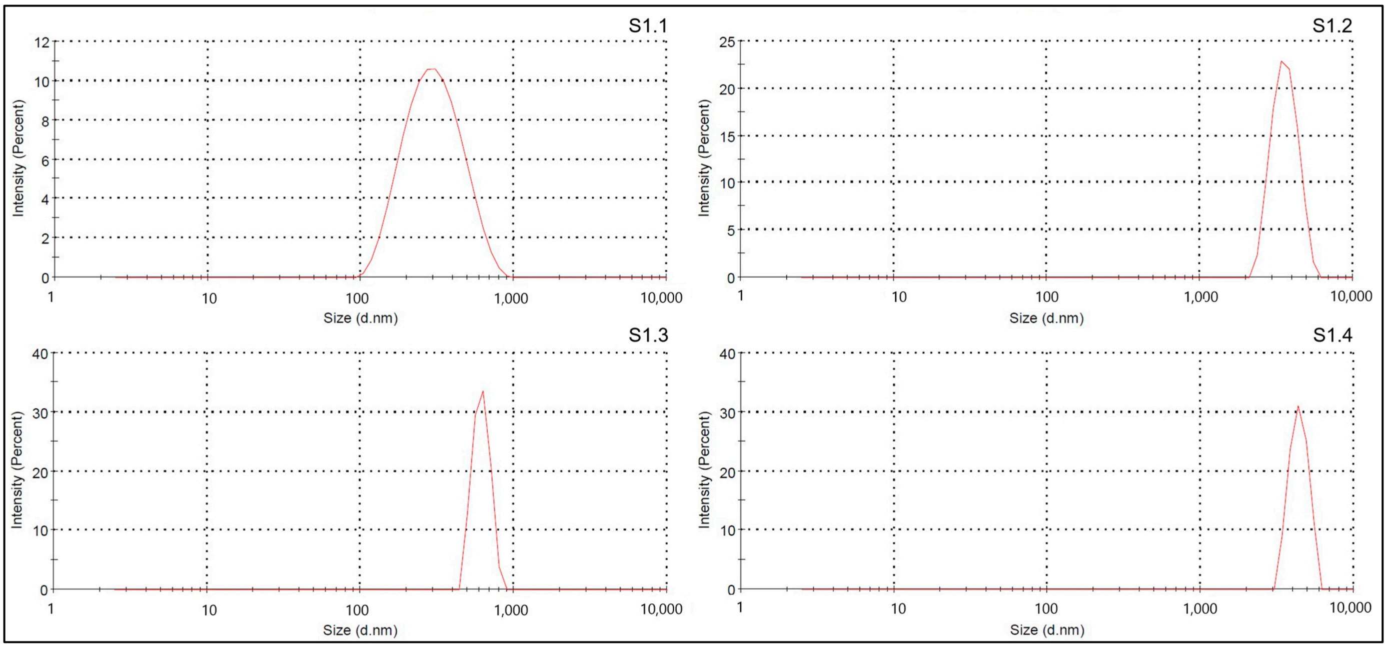

Figure S1. Particle size distribution of forced-degraded nab-paclitaxel 250 µg/mL; S1.1 heated sample, S1.2 sample degraded by acid plus heat, S1.3 sample degraded by base plus heat, S1.4 sample degraded by oxidation plus heat.

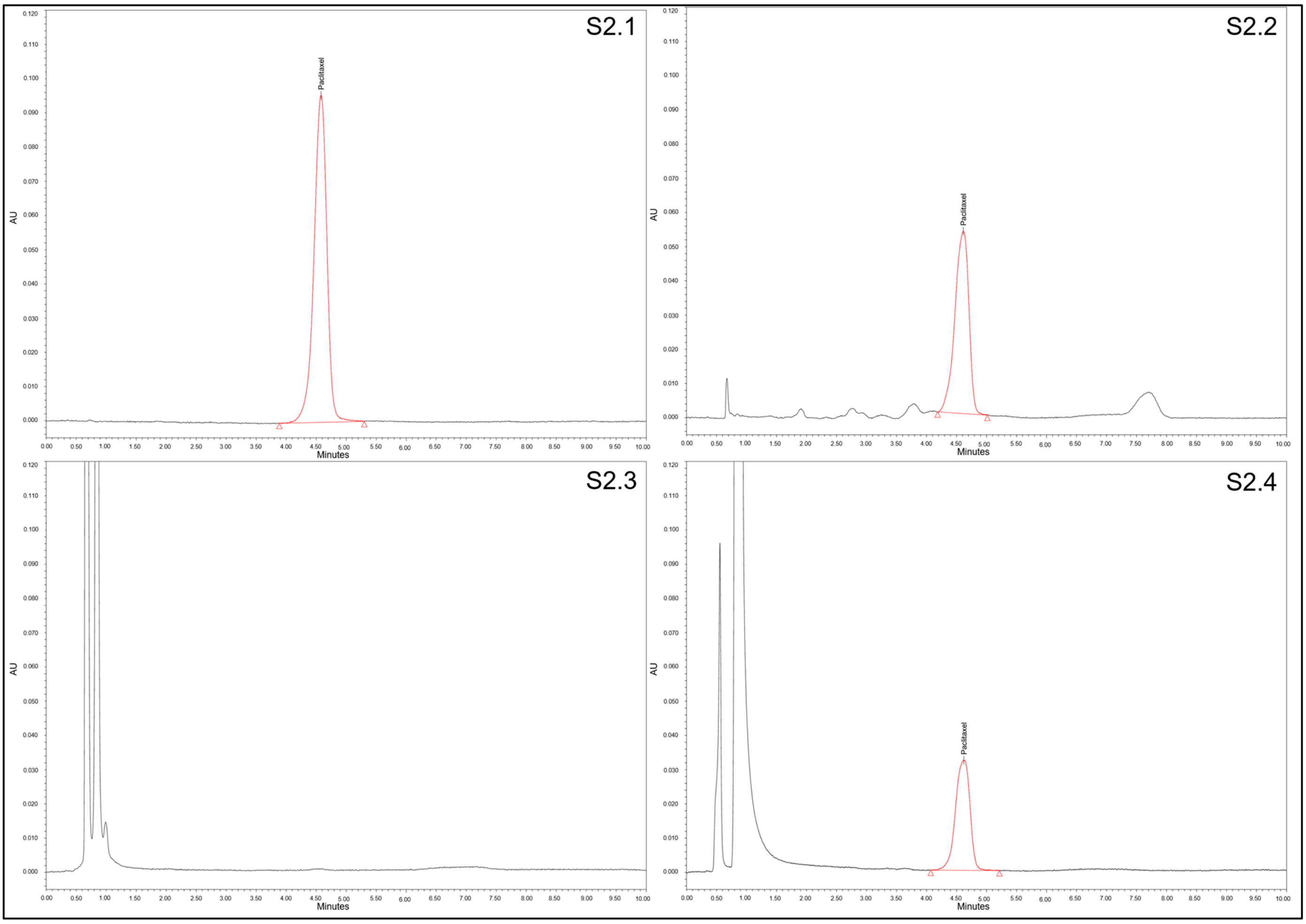

Figure S2. RP-HPLC chromatograms of forced-degraded paclitaxel CRS substance; S2.1 heated sample, S2.2 sample degraded by acid plus heat, S2.3 sample degraded by base plus heat, S2.4 sample degraded by oxidation plus heat.

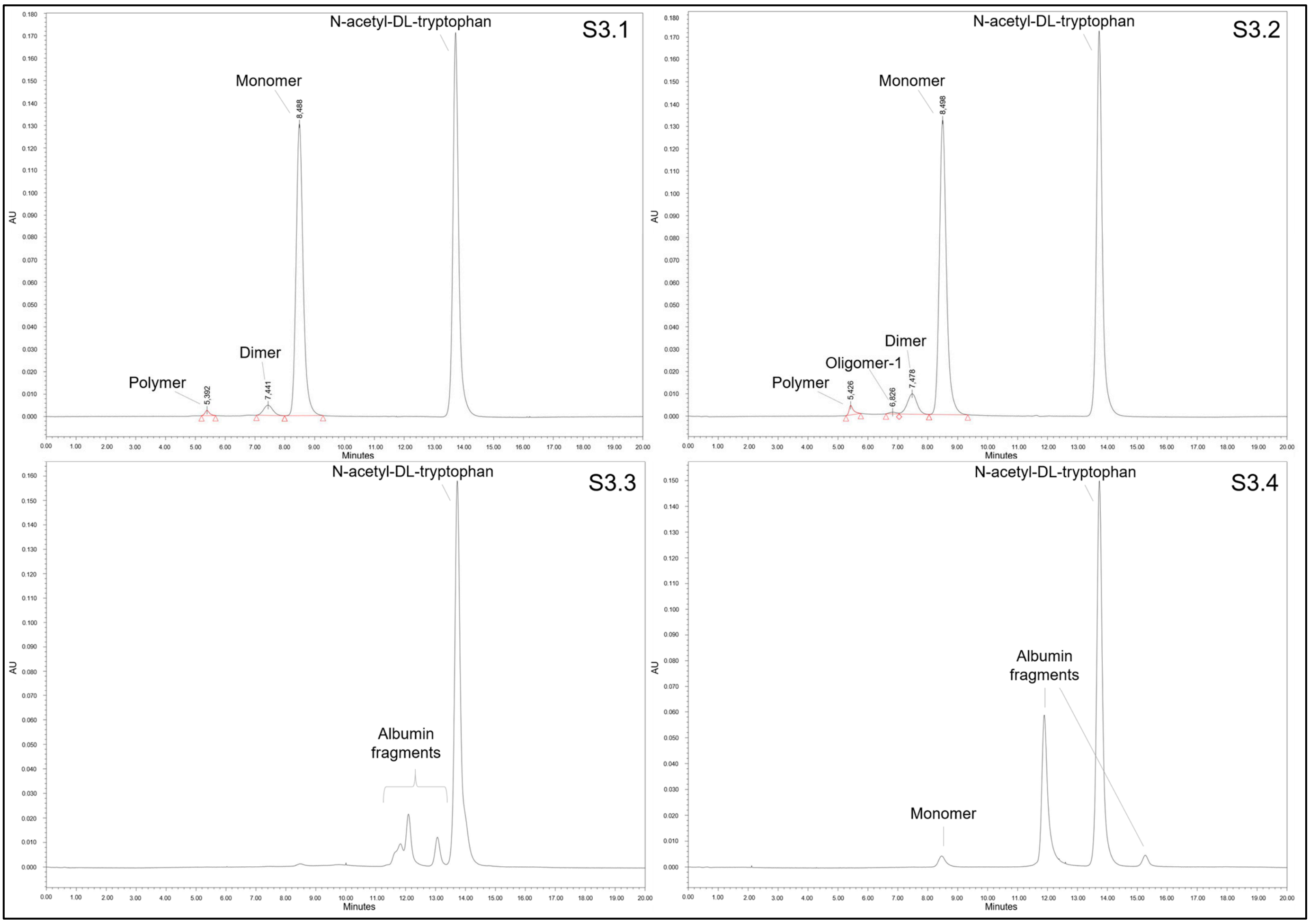

Figure S3. SEC chromatograms of forced-degraded nab-paclitaxel dilutions; S3.1 heated sample, S3.2 sample degraded by acid plus heat, S3.3 sample degraded by base plus heat, S3.4 sample degraded by oxidation plus heat.
